# Foam nest components of the túngara frog: a cocktail of proteins conferring physical and biological resilience

**DOI:** 10.1098/rspb.2008.1939

**Published:** 2009-02-25

**Authors:** Rachel I. Fleming, Cameron D. Mackenzie, Alan Cooper, Malcolm W. Kennedy

**Affiliations:** 1 Division of Ecology and Evolutionary Biology, Faculty of Biomedical and Life Sciences, University of GlasgowGraham Kerr Building, Glasgow G12 8QQ, UK; 2 WestChem Department of Chemistry, University of GlasgowJoseph Black Building, Glasgow G12 8QQ, UK

**Keywords:** túngara frog, *Engystomops pustulosus*, foam nests, lectins, surfactant protein, cystatin activity

## Abstract

The foam nests of the túngara frog (*Engystomops pustulosus*) form a biocompatible incubation medium for eggs and sperm while resisting considerable environmental and microbiological assault. We have shown that much of this behaviour can be attributed to a cocktail of six proteins, designated ranaspumins (Rsn-1 to Rsn-6), which predominate in the foam. These fall into two discernable classes based on sequence analysis and biophysical properties. Rsn-2, with an amphiphilic amino acid sequence unlike any hitherto reported, exhibits substantial detergent-like surfactant activity necessary for production of foam, yet is harmless to the membranes of eggs and spermatozoa. A further four (Rsn-3 to Rsn-6) are lectins, three of which are similar to fucolectins found in teleosts but not previously identified in a land vertebrate, though with a carbohydrate binding specificity different from previously described fucolectins. The sixth, Rsn-1, is structurally similar to proteinase inhibitors of the cystatin class, but does not itself appear to exhibit any such activity. The nest foam itself, however, does exhibit potent cystatin activity. Rsn-encoding genes are transcribed in many tissues of the adult frogs, but the full cocktail is present only in oviduct glands. Combinations of lectins and cystatins have known roles in plants and animals for defence against microbial colonization and insect attack. Túngara nest foam displays a novel synergy of selected elements of innate defence plus a specialized surfactant protein, comprising a previously unreported strategy for protection of unattended reproductive stages of animals.

## 1. Introduction

The use of foams as incubation environments for eggs or juveniles is widespread among animals, examples including the protective foam enclosures of the nymphs of homopteran bugs, bubble raft nests of fish and the egg chambers of locusts. In addition, recent work has described a marine biofoam that increases fertilization success and effective settlement of the short-lived larvae of tunicates (Castilla *et al.* 2007). Among the largest foam nests are those built by various tropical and subtropical frogs to contain their eggs and developing larvae. Depending on species, these are produced in underground burrows, suspended in vegetation or floating on the surface of water. Frogs present a particularly interesting case because this style of nesting appears to have evolved independently in several lineages (e.g. the Myobatrachidae of Australasia, Leptodactylidae of the Americas and the Rhacophoridae of Afro-Asia). The materials used for foam nest construction must be environmentally resilient, resistant to microbial assault, yet be compatible with naked eggs and sperm. The latter feature presents an interesting paradox because production of stable foams and bubbles requires surface-active, detergent-like compounds in order to facilitate foaming in water, whereas conventional, small molecule detergents would by their very nature be highly damaging to unprotected cell membranes and would destroy gametes upon contact.

The túngara frog of tropical America, *Engystomops pustulosus* (formerly *Physalaemus pustulosus*), presents a foam nesting strategy that is particularly precarious in both environmental and biological terms. The nests are produced at night, in water at the edges of puddles, ditches or other temporary standing water after rainfall (figure S1 in the electronic supplementary material). During mating, the female periodically releases eggs and foam precursor fluid, which the male gathers with his feet and, while releasing sperm, whips up with rapid movements of his legs to create a white foamy mass into which the eggs are incorporated. These nests, often in large communal masses, remain attached to adjacent soils or vegetation as water levels subside. Embryogenesis takes place over the next 2–3 days, and tadpoles are ready to leave the nest at approximately 3 days under normal conditions, though they can remain within the nest longer if water is not available (Downie 1993). In the absence of developing eggs or tadpoles, we have observed that the foams remain stable and intact for at least 10 days in tropical conditions, with only marginal dehydration and no sign of bacterial or fungal degradation. This is extraordinary, considering the microbial content of the waters with which these nests are constructed. The compatibility of the foam with eggs and sperm suggests that this resistance to microbial assault is not due to the membrane disruption expected of simple detergent activity, but is a more complex property of the foam components. These foams are remarkable biological materials whose structural properties, resistance to microbial attack and compatibility with sensitive gametes and developing embryos are yet to be explained.

In earlier work (Cooper *et al.* 2004), we have shown that the túngara frog nest foam fluid is made up of a dilute mixture (approx. 1–2 mg ml^−1^ each) of proteins and complex carbohydrates (as yet uncharacterized) with surfactant and other biophysical properties consistent with the accumulation of proteins at the air–water interface. We report here on a much more detailed biochemical characterization of the protein components showing that the foam comprises a small but remarkable set of proteins that includes an unusual protein surfactant, together with a combination of proteins consistent with anti-microbial and anti-insect protection not previously described in land vertebrates.

## 2. Material and methods

### (a) Nest proteins and partial sequencing

Amplexing pairs of *E. pustulosus* were captured at night in the northern mountain range of the Caribbean island of Trinidad, transferred temporarily to the laboratory at the Department of Zoology, University of the West Indies, St Augustine, Trinidad, placed in tanks of shallow, clean, dechlorinated water and allowed to nest overnight. The nest foam was manually separated to remove the eggs intact, and the foam stored frozen until use. Foam liquid was obtained either by centrifugation or sonication and separated on SDS–PAGE gels, stained for proteins and the six prominent bands excised and submitted for N-terminal amino acid sequencing by Edman degradation at the University of Leeds. Protein glycosylation was investigated using a commercial glycoprotein gel staining kit (Pierce Chemical, Rockford, IL).

### (b) cDNA isolation

After nesting, a sample (*n*=3) of female frogs was euthanized and the large foam-producing glandular section of the oviduct removed, immersed in RNAlater (Applied Biosystems, Warrington, UK) and stored at −20°C. Other tissues from both males and females were similarly sampled. N-terminal peptide sequences were used to design degenerate oligonucleotide primers for DNA sequencing (table S1 in the electronic supplementary material). Total RNA was isolated from the oviducts of breeding female frogs using the TRIzol reagent (Invitrogen Life Technologies, Paisley, UK) and the mRNA was reverse transcribed into cDNA using the Clontech (Basingstoke, UK) 3′ and 5′ RACE kit. Conditions for the 3′ RACE PCR reactions were 94°C for 3 min, followed by 30 cycles of 55°C for 30 s, 72°C for 60 s and 94°C for 30 s using a Biometra T-gradient thermal cycler. The 5′ RACE conditions were as described in the kit. PCR products were cloned into the TA sequencing kit for sequencing (Invitrogen) to obtain the full length sequences of all of the proteins, which were designated ranaspumins 1–6 (Rsn-1 to Rsn-6), with the GenBank accession codes AY226146 to AY226151. cDNA encoding β-actin was isolated by PCR using oligonucleotide primers designed around the known sequence from *Xenopus*, and is deposited in GenBank with accession code AY226144.

### (c) Amino acid sequence analysis

Sequence similarity searches using PSI-BLAST, and also those that use secondary structure predictions and fold recognition (including HHpred, Phyre and three-dimensional PSSM) were carried out via the ExPASy (www.expasy.org) servers, as were hydrophobicity plots (ProtScale set for the Kyte–Doolittle hydrophobicity scale with a nine-residue window; Kyte & Doolittle 1982), predictions for cleavable secretory signal leader peptides (SignalP; Emanuelsson *et al.* 2007) and searching for potential N-glycosylation sites (Prosite). Multiple alignments were produced by MultAlin (Corpet 1988) set for the Dayhoff evolutionary substitution matrix (Dayhoff *et al.* 1978). Protein structure modelling of Rsn-3, -4 and -5 was carried out using SwissModel, and fold recognition predictions by Phyre, both via the ExPASy website.

### (d) Recombinant proteins

PCR was performed on cDNA synthesized from oviduct mRNA, with gene-specific primers as listed in table S2 of the electronic supplementary material with the same conditions as for the 3′ RACE reaction (above). The PCR products were cloned into an Invitrogen Life Sciences pCR T7 TOPO TA Expression Kit with the plasmids transformed into BL21 (DE3) pLysS bacteria (Novagen/Merck Chemicals, Nottingham, UK). Bacteria were grown at 37°C and protein expression was induced using 1 mM isopropyl β-d-1-thiogalactopyranoside (IPTG). Recombinant proteins bearing histidine tags were purified by metal affinity chromatography using the His-Bind purification kit (Novagen). Protein concentrations were estimated from 280 nm absorbance using extinction coefficients calculated from amino acid compositions (ProtParam). Rsn-1 and Rsn-2 were purified under native conditions with further purification achieved using gel filtration on a Superdex 75 column (Amersham Pharmacia/GE Bioscience, Chalfont St Giles, Buckinghamshire, UK) using 50 mM Tris, 150 mM NaCl, pH 8.0. Rsn-4 and Rsn-6 were purified under denaturing conditions in the presence of 8M Guanidine–HCl (Gdn–HCl). The solubilized proteins were refolded by dropwise addition (approx. 1 ml h^−1^) to refolding buffer (100 mM Tris, 400 mM l-arginine, 50 μM oxidized glutathione, 0.5 mM reduced glutathione, 20 mM CaCl_2_ and 20 mM each of l-fucose, d(+)glucose (Fisher Scientific, Basingstoke, UK), maltose and galactose), totalling approximately 4 ml into 400 ml of refolding buffer, followed by overnight incubation, all at 4°C. The refolded proteins were concentrated using Vivaspin PES 20 ml centrifugal concentrators (MWCO 5000, Sartorius, Epsom, UK). Protein purity was routinely monitored by gel electrophoresis (SDS–PAGE) with Coomassie blue staining.

### (e) Agglutination assay

Haemagglutination activity of the putative lectins was determined by a serial dilution procedure using human erythrocytes. Each sample (20 μl, twofold serial dilutions in 10 mM HEPES, 150 mM NaCl and 2 mM CaCl_2_) was combined with 20 μl of a 2 per cent v/v suspension from packed cells in microtitre plates with U-shaped wells and allowed to sit for 1 hour at room temperature before reading. Binding specificity was tested by agglutination inhibition assays with inclusion in the buffer of 20 mM l-fucose, lactose, galactose, mannose, glucose, d(−) ribose and d(−) fructose (Sigma, Poole, Dorset, UK) as appropriate. Eel, *Anguilla anguilla*, fucolectin agglutinin (AAA) (Sigma) was used as a positive control.

### (f) Cystatin activity

Inhibition of proteolytic activity in a representative cysteinyl proteinase (papain) was investigated using fluorogenic or chromogenic substrates, Z-Phe-Arg 7-amido-4-methylcoumarin (Sigma) or Z-Phe-Arg-pNA (Bachem, St Helens, Merseyside, UK), respectively, using standard procedures (Morita *et al.* 1977; Tchoupe *et al.* 1991), at 30°C in 0.1 M acetate buffer, pH 5.0, containing 10 mM dithiothreitol and 2 mM EDTA. Reactions were initiated by adding papain (*Papaya latex*, Sigma) stock solution (0.5 U ml^−1^, in buffer) to substrate solutions (0.3–1 mM) in the presence or absence of putative inhibitors/control proteins (bovine serum albumin (BSA), lysozyme), and the relative rates of proteolysis measured from the linear increase in fluorescence (*λ*_ex_=370 nm, *λ*_em_=470 nm; Spex Fluoromax II) or absorbance (410 nm; Shimadzu UV-160) over a 2–3 min time period. Control experiments for serine proteinase (non-cystatin) inhibition were performed with trypsin/alpha 1-antitrypsin (Sigma).

### (g) Surfactant activity

Surface tension of aqueous solutions was measured at room temperature using a torsion balance (White Electrical Instrument Company) with a platinum Du Noüy ring. The ring was cleaned in detergent and rinsed exhaustively with deionized water, ethanol (HPLC grade, Fisher Scientific) and then flamed prior to measurement. Samples of 1 ml of foam fluid or recombinant protein solutions over a range of concentrations were allowed to equilibrate for 5 min before triplicate measurements. Experimental error for the surface tension was estimated by the standard deviation from the mean for each data point.

### (h) Sites of ranaspumin gene transcription

mRNA was isolated from a range of tissues as above. Gene-specific oligonucleotide primers (table S2 in the electronic supplementary material) were used in a conventional RT-PCR assay for transcripts that were detected by gel electrophoresis and ethidium bromide staining for amplicon DNA. Primers specific for *E. pustulosus* β-actin gene were used as positive controls for each tissue sampled, and DNA-free control reactions were all negative.

## 3. Results

Analysis of túngara foam nest material by gel electrophoresis (figure S2 in the electronic supplementary material) and subsequent cDNA sequencing revealed six abundant proteins in the 10–35 kDa range that we have named ‘ranaspumins’ (Latin: *rana*, frog; *spuma*, froth, foam), with designations Rsn-1 to Rsn-6. Parallel gel analysis of eggs separated from the foam showed that none of these proteins derived from disrupted eggs. Two-dimensional gel electrophoresis of the foam, together with mass spectrometry analysis of tryptic fragments, confirmed that Rsn-1 to Rsn-6 are the predominant proteins in the foam fluid (not shown). Partial amino acid sequencing of samples of each protein excised from gels were used to inform the design of oligonucleotides from which cDNAs encoding full coding regions were obtained. Resulting amino acid sequences are summarized in table 1, with full details deposited in GenBank. Gel staining for carbohydrate indicated no glycosylation of the natural ranaspumins, and no consensus N-glycosylation sites occur in their amino acid sequences. Molecular masses of proteins predicted from encoding cDNAs correspond adequately to their migration in SDS–PAGE gels.

The Rsn sequences obtained here have no direct or close matches in databases. Two of them are either of a novel type (Rsn-2) or only remotely similar to other classes of protein (Rsn-1), with the remainder showing partial similarity to previously reported proteins. However, guided by this sequence analysis, together with some biophysical intuition, we have been able to group the Rsns into three broad functional categories—carbohydrate binding, surfactant activity, and proteinase inhibition—relating to the structure and protection of these foam nests, and have tested this using recombinant proteins, where possible, as follows.

### (a) Carbohydrate binding

Rsn-3, -4, -5 and -6, which together comprise the bulk of the foam proteins, all have amino acid sequences resembling those of known carbohydrate-binding proteins, lectins. Rsn-3, -4 and -5 are similar to a family of fucose-binding proteins, the so-called ‘fucolectins’, which have been described as contributing to innate anti-microbial defence in blood, gills and eggs of teleost fish (Honda *et al.* 2000; Cammarata *et al.* 2001; Piskarev *et al.* 2003, 2004). Proteins of this family have not been found in any non-amphibian land vertebrate, but have been described from genomic or transcriptomic studies on the fully aquatic amphibian *Xenopus laevis* (Masse *et al.* 2004; see figure S3 in the electronic supplementary material for an alignment of Rsn-3, -4 and -5 with sample members of this family of fucolectins). Rsn-6 falls into a different category of lectins (C-type) that occur widely in nature, frequently associated with galactose binding, and it possesses a typical cleavable secretory signal sequence that is absent in the foam-derived protein.

**Figure 1:**
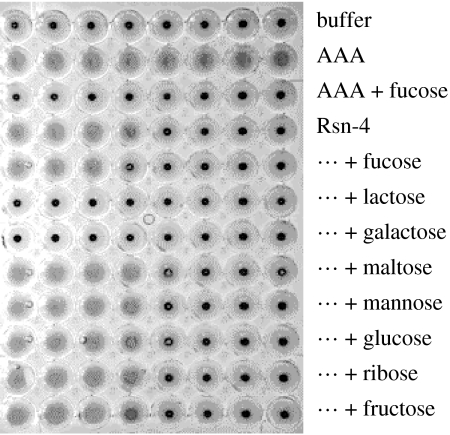
Lectin activity of Rsn-4. Agglutination of human red blood cells by recombinant Rsn-4 and AAA in the presence or absence of competing sugars. Positive agglutinations result in the formation of a sheet of cells over the base of the wells, negative reactions as buttons of cells in the centre. Note that, unlike the control fucolectin (AAA), agglutination by Rsn-4 was unaffected by fucose but completely inhibited by galactose or by the galactose-containing disaccharide, lactose. Other sugars showed no significant effects at this level. Similar results were obtained for cells of blood groups A, B or O.

The anticipated lectin activity was confirmed using recombinant Rsn-4, which agglutinated human erythrocytes of blood groups A, B or O (figure 1). This indicates that, as other lectins, Rsn-4 is able to form dimers or higher order complexes in order to cross-link carbohydrate-tagged particles. Interestingly, this activity was not inhibited by the addition of fucose, in contrast to the fucose inhibition easily demonstrable for the positive control, eel *A. anguilla* agglutinin (AAA, figure 1), which is the archetypical member of the teleost fucolectin family (Honda *et al.* 2000). Further studies using other competing sugars showed that Rsn-4 could be inhibited with lactose and galactose, but not maltose, glucose, ribose or fructose. Lactose is a disaccharide of galactose and glucose, so the specific sugar groups for Rsn-4 presumably contain galactose. Consequently, although demonstrably a lectin, Rsn-4 does not conform to the binding specificity expected of the family to which it belongs. Additional evidence for lectin activities was obtained by affinity chromatography with lactose–agarose columns followed by SDS–PAGE analysis of proteins eluted with 50 mM lactose, indicating that recombinant Rsn-4 and at least two proteins in natural nest foam bound, one of which is likely to be Rsn-4 itself (not shown). We found no erythrocyte agglutination with recombinant Rsn-3 or Rsn-6 under the same conditions, indicating that their carbohydrate-binding specificities are different from both Rsn-4 and AAA.

Sequence alignments of Rsn-3, -4 and -5 with other fucolectins show that they retain features that are conserved across the entire family, including all but one of the amino acid positions known to coordinate a single calcium ion (Bianchet *et al.* 2002; see also figure S4 in the electronic supplementary material). Protein structure predictions using the AAA lectin crystal structure (Bianchet *et al.* 2002) as a template, show that the Rsn-3, -4 and -5 sequences could all fold in a similar fashion (not shown). There are, however, differences in the amino acids that form the carbohydrate-binding site, as identified in the AAA structure, indicating that the Rsns are indeed likely to bind different sugars than the fucolectins previously described. These Rsns display other highly unusual features. Specifically, only in the Rsn-4 cDNA sequence, do we find the cleavable hydrophobic leader peptide expected for a secreted protein, the removal of which was confirmed by direct amino acid sequencing of foam-derived protein. By contrast, both Rsn-3 and Rsn-5 have moderately hydrophobic N-terminal sequences that are retained in the natural protein. This is highly unusual for secreted proteins, and particularly so for Rsn-3 for which the sequence is predicted by consensus to be a secretory signal that ought to have been removed prior to secretion. This unusual property could, however, relate to their association with an air–water interface, where the hydrophobic region might serve to anchor or orient the protein in the interface layer. Such behaviour would be unusual for lectins, but consistent with a special function in biological foam or air–water interface elsewhere in the adult frogs.

### (b) Surfactant activity

**Figure 2:**
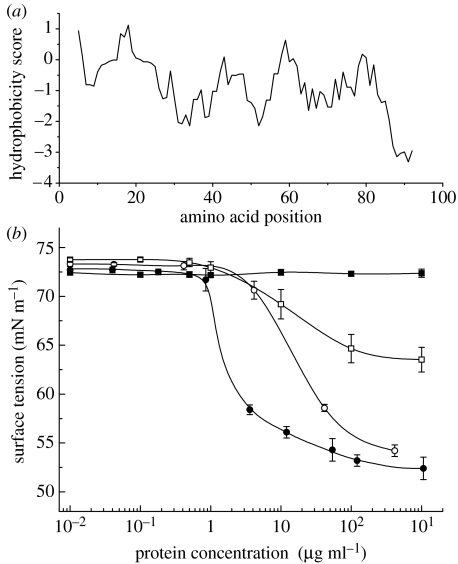
Unusual hydrophobicity profile and surfactant activity of Rsn-2. (*a*) Kyte–Doolottle hydropathicity plot of Rsn-2 illustrating the unusual hydrophobic N-terminal region of the mature protein and notably hydrophilic C-terminus. (*b*) Surface tension versus concentration of recombinant Rsn-2 (filled circles) in comparison with the natural foam fluid (unfilled circles) mixture, together with a negative control (lysozyme, filled squares) and a moderately surfactant control protein (bovine serum albumin; BSA, unfilled squares).

The amino acid sequence of Rsn-2 bears no similarity to any proteins presently in the available databases. The sequence shows an unusual amphiphilic distribution of amino acid residues such that the N-terminal region is relatively hydrophobic and the C-terminal region is markedly hydrophilic (table 1; figure 2*a*). The latter region notably contains a highly charged contiguous sequence of aspartic acid residues, contributing to an unusually high overall aspartic acid content (21.6% for Rsn-2 versus the SwissProt average of 5.3%). Such amphiphilic distribution, while lacking any obvious precedent in other proteins, is highly suggestive of potential surfactant activity. This was confirmed by surface tension measurements (figure 2*b*) showing that recombinant Rsn-2 substantially reduces the surface tension of water to a degree much greater than control proteins, and at a substantially lower protein concentration (1–10 μg ml^−1^) than the crude mixture from natural nest foam. A reduced surface tension is one of the necessary requirements for generation of foam in water, and whipped up samples of dilute Rsn-2 solutions do indeed produce foam superficially similar to the natural nest material. Unlike the foam in natural nests that are stable for many days, however, recombinant Rsn-2 foam collapses over a period of a few minutes, indicating that additional factors are required to explain the physical stability of the natural foam.

### (c) Proteinase inhibition

**Figure 3:**
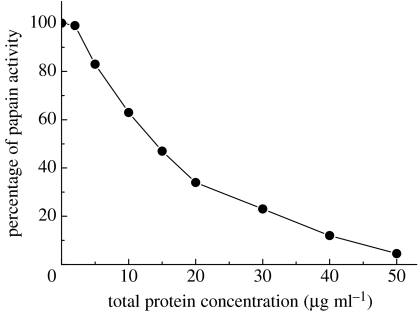
Potent proteinase inhibition by túngara frog nest foam. Inhibition of papain proteolytic activity by natural foam nest fluid mixture was measured as a function of total foam fluid protein concentration. Relative proteolysis rates determined at 30°C, pH 5.0, using the chromogenic peptide substrate, Z-Phe-Arg-pNA. Note that proteinase inhibition was essentially complete at 50 μg ml^−1^ total protein, which corresponds to more than 20-fold dilution of the natural material. Control proteins (BSA and lysozyme) showed no inhibition over the same concentration range. Error bars for each data point (from replicate measurements) are smaller than the data symbol used here.

Database searches with the amino acid sequence of the remaining protein, Rsn-1, revealed no matches other than remote similarities with known cysteinyl proteinase inhibitors (cystatins), see figure S5 in the electronic supplementary material. The predicted tertiary structure is also similar, but these similarities neither encompass the normally conserved functional regions of the cystatin family, nor did we find recombinant Rsn-1 to show any cystatin activity using either chromogenic or fluorogenic substrate assays. Plant seeds often have mixtures of lectins and cystatins as part of their protection against infection and insect attack. We therefore investigated túngara nest foam for cystatin activity using papain as the target enzyme and found that natural foam fluid exhibits potent cystatin activity (figure 3). We have, however, not so far been able to identify the component(s) in the foam responsible for this remarkable proteinase inhibition.

### (d) Sites of ranaspumin gene transcription

In order to establish whether the ranaspumins are specific to the foam nests or also occur elsewhere in the tissues of the adult frogs, we examined a range of tissues for mRNA encoding each of the Rsn proteins. This showed that all the ranaspumin genes were variously transcribed at other sites, but that the complete set was represented only in the glandular part of the oviduct that produces the foam precursor fluid (table 2). Interestingly, we found slight differences in the pattern of gene transcription between the tissues of individual frogs, which could indicate that ranaspumin synthesis may be inducible, depending on tissue type and the presence of infection or trauma. But, there were no exceptions to the rule that all of the ranaspumin genes were transcribed in glandular oviducts of all three females sampled.

## 4. Discussion

Water-based foams are inherently fragile and, especially in a biological context, must incorporate a range of functionalities. It is therefore satisfying to find that the cocktail of proteins identified here in túngara nest foam seems to fulfil many, if not all of the necessary prerequisites for biocompatibility and resilience. The first requirement in any aqueous foaming process (at least in dilute solutions) is to overcome the high-surface tension of water to facilitate bubble formation. Conventionally, this involves the use of small detergent molecules that would disrupt biological membranes (e.g. shampoos and soaps). Alternatively, foams may be formed by vigorous mechanical agitation of highly viscous solutions, usually requiring concentrations of proteins (often denatured) or other polymers much higher than observed here (cf. egg white/meringue, with a protein/ovomucoid concentration usually exceeding 90 mg ml^−1^; Powrie 1973). Interestingly, although we have observed a much more viscous consistency in foam nests of other species (e.g. *Polypedates leucomystax*; R. Tan, A. Cooper & M. W. Kennedy 2004, unpublished observations), such a strategy would seem to be expensive in terms of protein synthesis and the mechanical energy required for nest construction. Rsn-2 plays the surfactant role more economically here at very much lower concentrations (approx. 0.1 mg ml^−1^), while avoiding membrane disruption, thus resolving the curious paradox that, while the foam exhibits a necessary detergent-like surface activity necessary for the very existence of a foam, it is at the same time harmless to eggs and sperm. We have found no evidence for small molecule detergents in túngara foam, so the key to the problem must reside in the particular properties of the surfactant protein itself. It is possible that the vitelline membrane or a thin jelly layer, both of which may exclude a surfactant protein, protects the eggs, although spermatozoa would not be similarly protected. The conformation of Rsn-2 might therefore be such that it does not insert into and damage eukaryotic membranes. This is most likely because the amphiphilic nature of the macromolecule allows incorporation at the air–water interface in foam bubbles, while its size prevents the infiltration into lipid bilayers and subsequent disruption seen with conventional detergents. This is consistent with our observation made during our agglutination experiments that no cell lysis occurs when foam nest material is mixed with mammalian erythrocytes.

The best understood macromolecular biological surfactants are those of the vertebrate lung, but these are associated with lipids. Recombinant Rsn-2, on the other hand, exhibits strong surface activity, so this property cannot be associated with lipids or carbohydrate and must therefore be intrinsic to the protein itself. This is also true of only a few other proteins known, such as the latherin protein of horse sweat (Beeley *et al.* 1986) and the hydrophobins of fungi (Hakanpaa *et al.* 2004), neither of which show any amino acid sequence similarity to Rsn-2. In the case of hydrophobins, the structure of the protein reveals distributions of hydrophobic and charged amino acids on the surface of the proteins that probably explain their surface activity (Hakanpaa *et al.* 2004).

**Figure 4:**
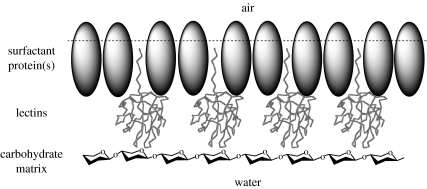
Cartoon depicting the possible arrangement of ranaspumins and complex carbohydrates at the air–water interface. Surfactant proteins (predominantly Rsn-2) perform the primary role of surface tension reduction to allow initial bubble/foam production. This layer may then be further stabilized by incorporation of lectins (e.g. Rsn-3, Rsn-5 via their hydrophobic tails) to which the long-chain, branched polysaccharide components of the natural foam fluid may attach. This would create a mechanically stable, water-retaining foam matrix.

Other proteins in the mixture may also have surfactant roles (e.g. Rsn-3 and Rsn-5) since they possess relatively hydrophobic N-terminal regions in their secreted forms, and may be involved in the structure of the foam itself. While none of the ranaspumins are themselves glycosylated, approximately half of the non-water mass of the nest material is carbohydrate (Cooper *et al.* 2004) to which the lectins may bind, cross-link and thereby stabilize the structure of the foam. Added to this is the retention of hydrophobic leader peptides in Rsn-3 and Rsn-5, which might indicate association with the air–water interface, and we already know that foam proteins accumulate there (Cooper *et al.* 2004). It is therefore conceivable that these lectins may associate at the interface and participate in a cross-linked matrix that greatly extends the lifetime and stability of these foams (figure 4). Our biophysical analysis of the natural foam mixture indicated that initial foam formation is facilitated by a specific protein (now shown here to be Rsn-2), but is then further stabilized by subsequent aggregation and cross-linking of other components to form a multilayer surface complex (Cooper *et al.* 2004). This resolves the apparent paradox that, although Rsn-2 is a very effective surfactant, but, as conventional soaps and detergents, by itself it produces relatively short-lived foam; combination with other foam components (lectins and carbohydrates) leads to synergistic long-term stabilization. The carbohydrate-binding activity of the lectins could also act to tether the lectins in place of the foam and prevent their loss from the nest by simple diffusion into the underlying water, and complex carbohydrates would be very effective in retaining water and restraining dehydration.

The predominant role of the majority of ranaspumins (Rsn-3, -4, -5, -6 lectins) and the unidentified cystatin(s) is probably in defence against pathogens or parasite attack. Lectins may have several functions, the most immediate being to counter microbial infections. Such a function would be particularly pertinent to túngara frogs because they construct their nests using pond water that contributes most of the mass of the nests and is heavily contaminated with microbes. Lectins occur elsewhere in sensitive tissues of animals and plants (invertebrates, fish gills, blood and eggs; mammalian lungs; plant root tips, seeds and bulbs; Honda *et al.* 2000; Holmskov *et al.* 2003; Van Damme *et al.* 2004; Iwanaga & Lee 2005; Gupta & Surolia 2007; Chen 2008), and as generic microbe recognition components of the vertebrate immune system (Gupta & Surolia 2007; Van Kooyk & Rabinovich 2008). Lectins bind to microbial surfaces but are unable to kill without accessory proteins, although they can agglutinate particles bearing their target sugars and thereby impede dissemination. Their role in frog nest foams may therefore be to restrain microbial colonization of the foam and eggs and to disable cell surface receptors and nutrient transporters of invading pathogens; it is already known that fungal attack is a particular risk to aquatic frog eggs (Gomez-Mestre *et al.* 2006, 2008; Touchon *et al.* 2006). So far, we have found no evidence in the foam of anti-microbial peptides such as the magainins of adult frogs, so the lectins may bear the main burden of protection.

Three out of the four lectins found in túngara nest foam have amino acid sequences similar to a family of fucose-binding lectins (fucolectins) originally described from teleost fish (Honda *et al.* 2000; Cammarata *et al.* 2001; Piskarev *et al.* 2004). Similar amino acid sequences have been found in *Xenopus* spp. (Kakinuma *et al.* 2003; Masse *et al.* 2004; Ishino *et al.* 2007), but not in fully adapted land vertebrates. The fact that Rsn-4 does not bind fucose, but rather lactose and galactose, indicates that this lectin family may have a much broader range of specificities than previously realized, suggesting that amphibian versions have diversified for the particular infection challenges that they face.

Lectins will also probably play a role in protection against parasitism or predation of the eggs. Elsewhere, it has been shown that lectins found in some plant tissues and seeds are severely damaging to the gut tissues of insects and vertebrates, so are thereby protective against predation (Zhu-Salzman *et al.* 1998; Murdock & Shade 2002; Sauvion *et al.* 2004; Dutta *et al.* 2005; Hossain *et al.* 2006; Chen 2008), possibly by interfering with the repair of vulnerable absorptive epithelial cell of the (mammalian) gut (Miyake *et al.* 2007). For frogs, parasitism by several species of dipteran (‘frog flies’) that specialize in laying their larvae on frog foam nests is a particularly important risk factor, from which complete loss of the brood is common (Downie *et al.* 1995; Menin & Giaretta 2003; M. W. Kennedy 2004, unpublished observations), up to 30 per cent of frog nests can be destroyed in this manner (Littlejohn *et al.* 1993).

Inhibition of proteolysis is another potential defence strategy, and inhibitors of cysteinyl proteinases (cystatins) have been shown to be effective against parasites or predators, as exemplified by the role of plant cystatins in the defence against attack by insects and nematodes (Blankenvoorde *et al.* 1998; Koiwa *et al.* 2000; Cooper *et al.* 2004; Haq *et al.* 2004; Outchkourov *et al.* 2004; Khaithong *et al.* 2005; Lalitha *et al.* 2005; Chen 2008). In addition to the túngara nests described here, we have also found potent cystatin-like activity in unfractionated foam nests of other species (*Rhacophorus arboreus*, *P. leucomystax*; R. Tan, A. Cooper & M. W. Kennedy 2006, unpublished data). It is therefore likely that the lectins and cystatin(s) may act together against predation or parasitism. In addition, as recently proposed elsewhere in a different context (Rostás & Blassmann 2009), the intrinsic surfactant activity of the foam might itself serve as a defence against insect attack.

The samples we investigated here came from the Caribbean island of Trinidad, but we have also examined túngara nest material from a Panamanian population and found no difference in the protein profile, although we do not yet know whether any sequence variants exist. We have also examined foam of other foam nesters, *Leptodactylus fuscus* of Central and South America, the Australasian *Limnodynastes peronii* and the Asiatic aerial-nesting species *P. leucomystax* and *R. arboreus*, and found each to have a distinct profile of proteins (figure S6 in the electronic supplementary material). Likewise, neither the recently reported partial amino acid sequence of a protein from *Leptodactylus vastus* foam nests (Hissa *et al.* 2008) nor our own complete sequence of a protein from the nests of *L. fuscus* (GenBank AY560613) bear any resemblance to the ranaspumins reported here. Pertinent to this diversity, we have recently shown that the unrelated frog nest foam of *P. leucomystax* contains a blue-green protein exhibiting a chromophore and protein–protein cross-link unlike any previously observed in nature (Oke *et al.* 2008). The physical consistency of nest foams also varies dramatically between species, from wet foams such as that of the túngara frog, to the stickier, viscous foams of those that attach their nests to vegetation, and this may also contribute to the diversity of nest constituents.

It is noteworthy that transcription of none of the genes encoding Rsns is exclusive to the foam-producing tissue. Rather, the proteins are differentially expressed in different tissues of the frogs (assuming gene transcription leads to protein in those tissues). It could therefore be that the Rsns did not evolve primarily for construction and protection of the nest, but were drawn from cell-compatible proteins that were already components of innate defences or adapted from other functions.

The features of túngara frog nest foam reported here show that the foam is not only a remarkable biomaterial, containing proteins with interesting new properties, but also that it shows aspects of convergent evolution for the defence of reproductive and dispersal stages of a wide range of organisms, from amphibians, to fish, to plants. The diversity of frog nest foams is therefore of intrinsic interest, not least for the general principles it may illuminate in terms of the evolution and adaptability of these unusual biological materials.

## Figures and Tables

**Table 1:** Nest foam protein amino acid sequences and similarities. (Ranaspumin complete amino acid sequences from direct partial amino acid sequencing of proteins purified from foam nests and subsequent cDNA analysis. Single-letter amino acid code. The underlined amino acids are those predicted by the SignalP algorithm set for eukaryotes to be secretory signal peptides that are normally removed before release from the synthesizing cell. Lower case letters refer to amino acids not found in the N-terminal sequence of the natural, foam-derived protein. The amino acid in bold uppercase is the first amino acid recorded by direct N-terminal amino acid sequencing of purified foam proteins.)

Rsn-1	sequence similarity: inhibitors of cysteinyl proteinases (cystatins)
maaiqfalffvfavisqhcaygflpl**G**GGNIGGGAKLGPEKPATPGIQDLLKSLLSVLNLSPPAIPEDAEAVSYRDAKNGKFRLIKIHLGGELYCHVKQIAGPILALPIVSDVVEVTGKECGKTEDDPLEDFPIP	
Rsn-2	sequence similarity: none found
m**L**ILDGDLLKDKLKLPVIDNLFGKELLDKFQDDVKDKYGVDTKDLKILKTSEDKRFYYVSVDAGDGEKCKFKIRKDVDVPKMVGRKCRKDDDDDDGY	(no secretory leader peptide, yet found as a secreted product in the nest foam)
Rsn-3	sequence similarity: fucolectins from teleosts
m**I**DPTGLVQILLLEQVVHKIPPGNINLARTGIATQDSDYTASAVPSEARLAIDGNRNSDFNQKSCSHTGGNEPAWWRLELKKKSKISVVVIAIRSDCCMDRFKGAELRIGNSQDATVNPICGKVSAVKGSNYLFCCDGMEGKYISVVIPDRHEFLSLCEVEVYGAKPIEGTHCK	(no secretory leader sequence, yet found as a secreted product in the nest foam)
Rsn-4	sequence similarity: fucolectins from teleosts
mkllllvvlvwtasdeasa**D**RNLALDGRATMSSIWMDPDIRQSFLGVAMNGIDGNTDSVYFHGSCFHTGLDSPAWYRVDLLRTSKISSITITNRGDFGSRTNGAEIRIGDSLANNGNNNPRCALVTSIADGETRTFQCNNMVGRYVNIVLTGKTEFLHLCEVQIFGENLPRSFSCQYSNDGMITLLVSTRFMK	
Rsn-5	sequence similarity: fucolectins from teleosts
m**G**APGGAAGPLLVLNILGSVVHETKPPEGVNLALKGIASSDSIASNGSVTGLAAKAIDGIRVSDFFKGHCSLTNGLNNPTWWKVDLKKSYKISSVFVTNRDDCCTERLLHAEIRIGSNPDHNHNPICAEVKTVASSNIGFCCGGMEGRYVSVSVPRKEQLSLCEVEVYGDLKKVLHCA	(note that the predicted hydrophobic secretory leader sequence persists in the intact foam-derived protein)
Rsn-6	sequence similarity: C-type, galactose-binding lectins
mililgvlllgaeasa**E**TLCIPGRMKQLDAGAGRVVAVKSNGDVYQLLENNWVQIPGKLIHVTVGPAGLWGVNKDKNIYKYVDNDWLQVDGLLNQIDAGGNRFVVGVNDNEDIFCLNQDQTTSNAVKLDYKGVDGKLKYYSSGGYGSWGVNAAYDIFYRRNVHPMSCQGTNWENVEGKLVMLEVAEDGSVYGVNYNGHVYKREGITAGNPMGTSWTYLKVDEKVRHVSYDRGVLYVVTIDDRIFRC	

**Table 2:** Tissue distribution of foam protein gene transcription in adult *E. pustulosus* frogs. (mRNA was isolated from tissues of three female and three male *E. pustulosus* and transcripts encoding each of the ranaspumins detected by RT-PCR as described in §2. Tissues in which transcripts were detected are indicated by a large black dot and those in which none were detected are left blank. The pattern varied slightly between the animals examined, positives ascribed if transcript was detected in at least two of the animals from each sex.)

tissue	Rsn-1	Rsn-2	Rsn-3	Rsn-4	Rsn-5	Rsn-6	β-actin
oviduct	•	•	•	•	•	•	•
ovary		•		•	•		•
liver		•		•			•
kidney		•		•	•		•
oesophagus				•			•
small intestine				•	•		•
large intestine			•	•	•	•	•
tongue			•				•
eye	•				•		•
heart					•		•
leg muscle					•		•
testis		•		•	•	•	•
lungs				•	•		•

## Supplementary Material

Additional tables, figures and references Oligonucleotides used, protein sequence comparisons and analyses, nest site habitat pictures, foam nest protein profiles from different species
