# Supplementary material for: Foam nest components of the túngara frog: a cocktail of proteins conferring physical and biological resilience
Source: Proc Biol Sci. 2009 Feb 25;276(1663):1787–95. doi: 10.1098/rspb.2008.1939 (PMC2674504; doi:10.1098/rspb.2008.1939)
Supplement: Additional tables, figures and references — Oligonucleotides used, protein sequence comparisons and analyses, nest site habitat pictures, foam nest protein profiles from different species [file rspb20081939s10.doc]

**Foam nest components of the túngara frog: a cocktail of proteins conferring physical and biological resilience**

Rachel I. Fleming^1^, Cameron D. Mackenzie^2^, Alan Cooper^2^ and Malcolm W. Kennedy^1^*

*^1^Division of Ecology and Evolutionary Biology, Institute of Biomedical and Life Sciences, Graham Kerr Building, and ^2^WestChem Department of Chemistry, Joseph Black Building, University of Glasgow, Glasgow G12 8QQ, Scotland, UK*

*Author address for correspondence: Division of Environmental and Evolutionary Biology, Graham Kerr Building, University of Glasgow, Glasgow G12 8QQ, Scotland, UK ([malcolm.kennedy@bio.gla.ac.uk](mailto:malcolm.kennedy@bio.gla.ac.uk)).

#### Electronic Supplementary Material

**Supplementary Table S1**. Oligonucletide primers used to isolate full-length cDNAs encoding each of the six foam proteins.

**Supplementary Table S2**. Gene-specific oligonucleotide primers used both to produce cDNAs for insertion into protein expression plasmids and to detect transcription of genes encoding each of the ranaspumins by RT-PCR.

**Supplementary Figure S1**. *E. pustulosus* nest sites and nests.

**Supplementary Figure S2**. Protein gel electrophoresis showing the major protein components of *E. pustulosus* nest foam.

**Supplementary Figure S3**. Similarities between Rsn-3, 4 and 5 and proteins of the fucolectin family.

**Supplementary Figure S4.** Similarities and differences between the sequences of the *A. anguilla* agglutinin (AAA) fucolectin and the fucolectin-like Rsn-3, -4 and -5 in terms of the amino acids known to interact with a calcium cation or fucose in the known 3-D crystal structure of AAA

**Supplementary Figure S5**. Similarities between Rsn-1 and cystatins (inhibitors of cysteinyl proteinases).

**Supplementary Figure S6.** Diversity in the protein profiles of frog foam nests.

**References for Electronic Supplementary Material**

**Supplementary Table S1**. Oligonucletide primers used to isolate full-length cDNAs encoding each of the six proteins.

The following primers were used to amplify cDNAs synthesised from mRNA isolated from the glandular region of the oviduct of female *E. pustulosus* after nesting. The gene-specific internal sequences so produced were then used to obtain full-length cDNAs by 5’ and 3’ RACE (see Materials and Methods).

| N-terminal amino acid sequence used for primer design | 3’ degenerate primer (3’–5’) | 5’ primer (3’ – 5’) |
| --- | --- | --- |
|  |  |  |
| Rsn-1  GGGNIGGGAKLGPE | ggnggnggnaayathggngg | ggactgagattcagcaca |
| Rsn-2  LDGDLLKDKLKLPVID | ctgctgaaagayaaaytyaaactg | ctaatatccatcatcatcatc |
| Rsn-3  ATRRLVRGGGG | gtctccatagacctcgacc | gggtggcgattccagtcctt |
| Rsn-4  DRNLALDGRATMSSIW | actatgwsnwsnatctggatg | ggccggagaatccaggccagtgtg |
| Rsn-5  GAPQSAAGPLLVLNIL | gcygcyggsccwctgctgrttskg | tcaggcacaatggaggactttttt |
| Rsn-6  ETLXXEIPGRMKQLDAG | atcccwggscggatgaag | attcagtaacccgtccacctgcag |
| β-actin | cagmtcacmgawgaygatat | ttagaagcatttrcggtggac |

For degenerate primers, the alternative nucleotide codes apply:

R = A + G; Y = C + T; M = A + C; K = G + T; S = G + C; W = A + T; H = A + T + C

N = A + G +C + T

**Supplementary Table S2**. The following gene-specific oligonucleotide primers were used both to produce cDNAs for insertion into protein expression plasmids in *E. coli* and to detect transcription of genes encoding each of the ranaspumins by RT-PCR in different tissues of adult *E. pustulosus*.

|  | Forward Primer (3’ – 5’) | Reverse Primer (3’ – 5’) |
| --- | --- | --- |
| Rsn-1 | ggtggtggtaatatcggtggtggt | tcagggaatggggaaatcctc |
| Rsn-2 | ttagatggggacctactaaaggacaagtta | ctaatatccatcatcatcatc |
| Rsn-3 | attgatccgaccggtctt | ctatttacaatgtgtcccttcaat |
| Rsn-4 | gatcgtaatctggcgctggacggtcga | ttatttcatgaatcttgtagatacaagtaaagt |
| Rsn-5 | ggggctcctggaggtgctgctggt | tcaggcacaatggaggactttttt |
| Rsn-6 | gaaactctgtgccttgagattcct | ttaacatctgaagattctatcatcaat |

### A


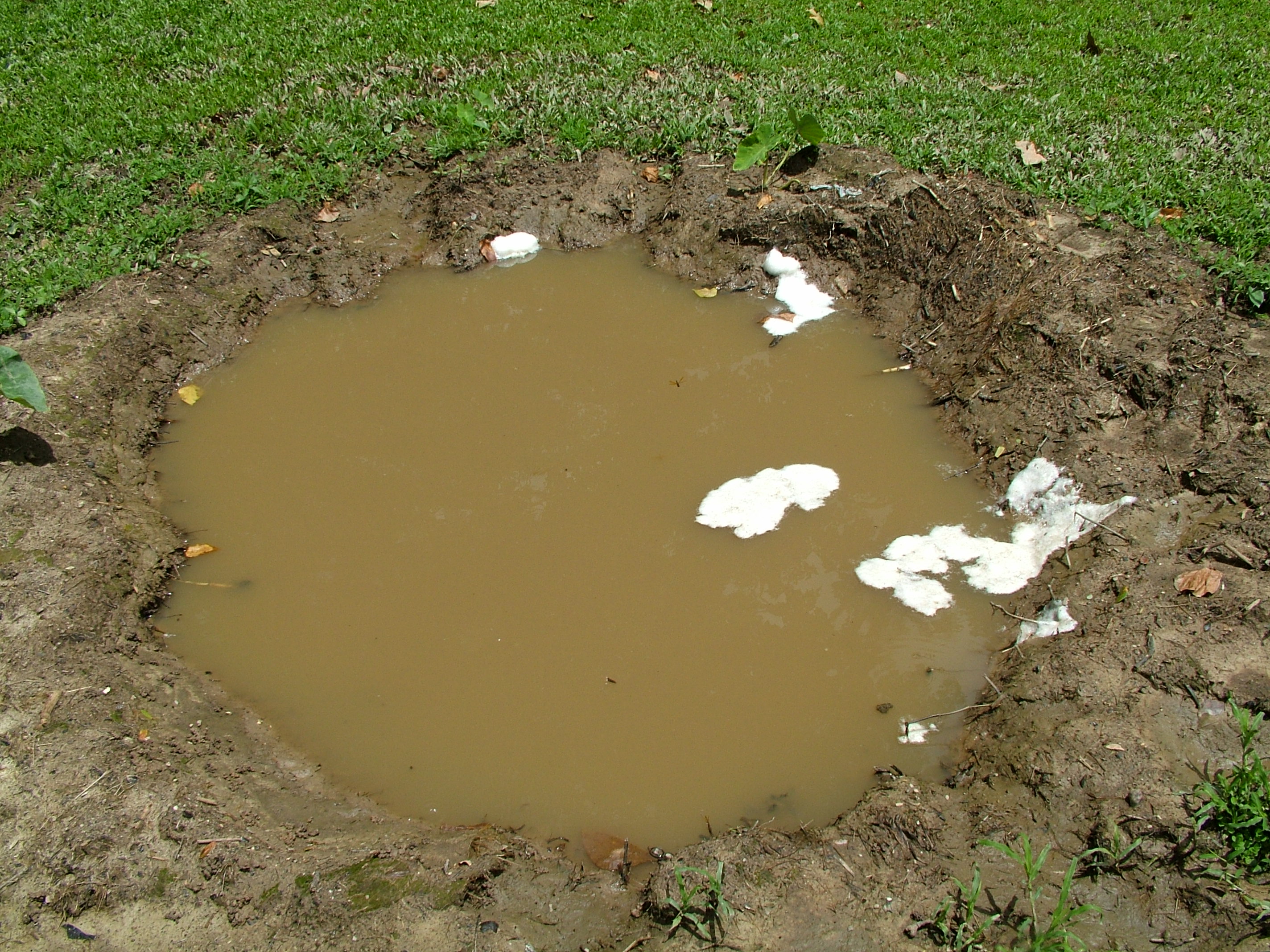


**B**


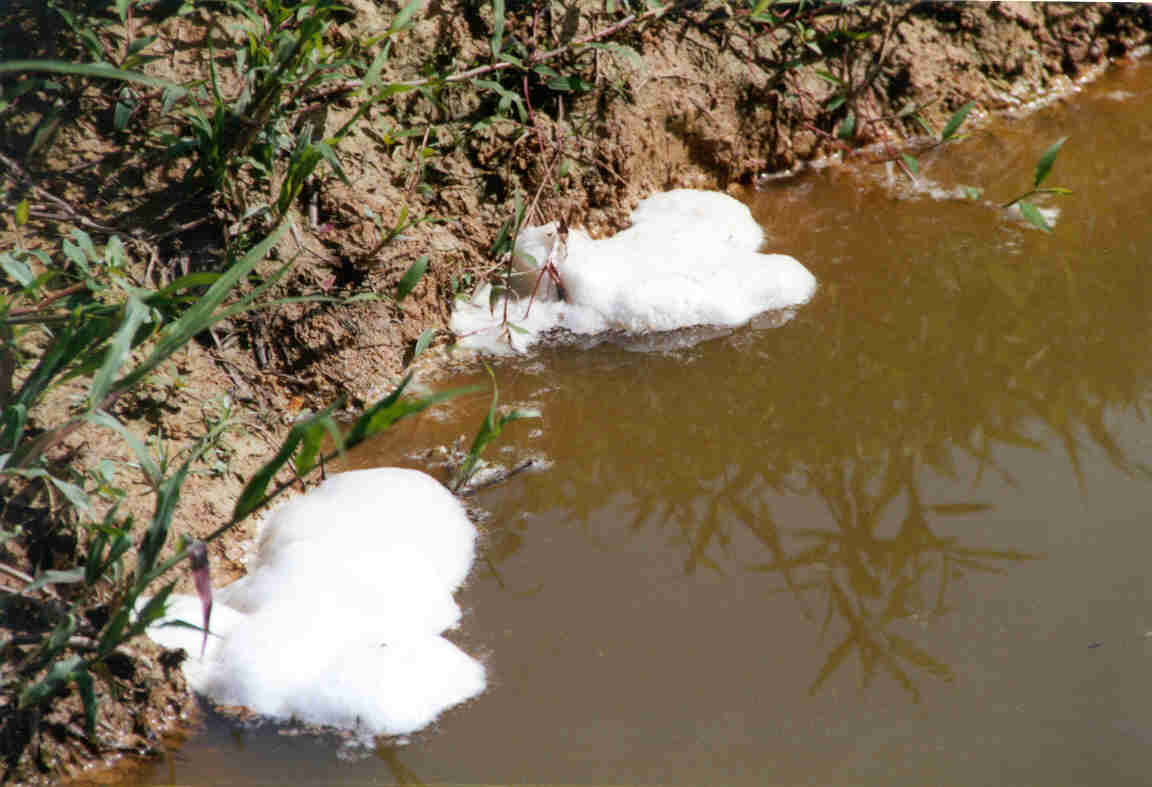


**Supplementary Figure S1**. *E. pustulosus* nest sites and nests illustrating the highly microbially contaminated water incorporated into the nests. (A) Several nests, some colonial, in a water buffalo pond approximately 1.5 m in diameter. (B) Close up of colonial nests. Individual nests are approximately 10 cm in diameter.

**Supplementary Figure S2**. Protein gel electrophoresis showing the major protein components of *E. pustulosus* nest foam. SDS-PAGE analysis of unfractionated nest foam fluid (3 μl) together with standard molecular mass reference proteins (track R), whose sizes are given in kiloDaltons (kDa). Stained for proteins with Coomassie Blue.

**Supplementary Figure S3**. Similarities between Rsn-3, 4 and 5 and proteins of the fucolectin family. One sequence from each of a teleost (the European eel, *Anguilla anguilla*, SwissProt Q7SIC1) and an amphibian (the African clawed toad, *Xenopus laevis,* Q64GD1) are aligned with the sequences of the three ranaspumins*.* The sequences were edited to remove highly divergent flanking regions and aligned using the Dayhoff evolutionary substitution matrix (Dayhoff et al. 1978). The single letter code for amino acids is used. The consensus line indicates where amino acid positions are conserved and contains indicators of where amino acids are of a similar type, in this case # is any one of D, E, N, or Q. Absolutely conserved positions are shown in red, those where an amino acid is conserved in the majority of sequences are in blue. Some of the amino acid positions known from the one fucolectin for which structural information is available (*A. anguilla* agglutinin; (Bianchet et al. 2002)) to be involved in binding of fucose are different in the Rsn proteins, indicating likely differences in their binding specificities for glycans – see below in Supplementary Figure S4.

1 50

AAA I**D**G**N**RDSN**FY** HG**S**CT**H**SSG. QANPWWRVDL LQVYTITSVT ITN**R**GD**CC**GE

Rsn-4 I**D**G**N**TDSV**YF** HG**S**CF**H**TGL. DSPAWYRVDL LRTSKISSIT ITN**R**GD**F.**GS

Rsn-3 I**D**G**N**RNSD**FN** QK**S**CS**H**TGG. NEPAWWRLEL KKKSKISVVV IAI**R**SD**CC**MD

Rsn-5 I**D**G**I**RVSD**FF** KG**H**CS**L**TNGL NNPTWWKVDL KKSYKISSVF VTN**R**DD**CC**TE

51 100

AAA **R**ISGAEINIG QHLASNGVNN PECSVIGSMA TGETKTFHCP APMIGRYVVT

Rsn-4 **R**TNGAEIRIG DSLANNGNNN PRCALVTSIA DGETRTFQCN N.MVGRYVNI

Rsn-3 **R**FKGAELRIG NS..QDATVN PICGKVSAVK GSNY.LFCCD G.MEGKYISV

Rsn-5 **R**LLHAEIRIG SN..PDHNHN PICAEVKTVA SSNI.GFCCG G.MEGRYVSV

101 117

AAA YLPT.SESLH L**CE**VEVN

Rsn-4 VLTGKTEFLH L**CE**VQIF

Rsn-3 VIPDRHEFLS L**CE**VEVY

Rsn-5 SVPRKEQ.LS L**CE**VEVY

**Supplementary Figure S4.** Similarities and differences between the sequences of the *A. anguilla* agglutinin (AAA) fucolectin and the fucolectin-like Rsn-3, -4 and -5 in terms of the amino acids known to interact with a calcium cation or fucose in the known 3-D crystal structure of AAA ((Bianchet et al. 2002)). The four fucolectin-type proteins edited and aligned as for Supplementary Figure S3 above. Amino acid positions aligning with those identified in the structure of AAA as interacting with the calcium cation found in AAA are indicated in blue, and those aligning with sugar-interacting amino acids in AAA are in red.


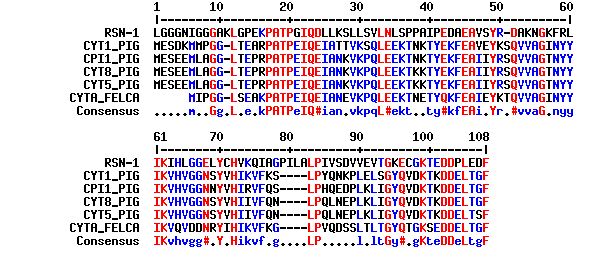


**Supplementary Figure S5**. Similarities between Rsn-1 and cystatins (inhibitors of cysteinyl proteinases). Sequence similarity searching was carried out by WU-BLAST search of the SwissProt database set for the same evolutionary matrix as above, and collecting only sequences providing probability E-values of values of 5.0e-6 or less. The SwissProt codes for the sequences below RSN-1 are, in order, Q28988, P35479, Q8WNR9, Q28986 and Q8WNR9. The alignment was also carried out as above. The fold prediction and recognition program Phyre ([www.sbg.bio.ic.ac.uk/~phyre/](http://www.sbg.bio.ic.ac.uk/~phyre/)) predicted that Rsn-1 may fold to form a 3-D structure like the cystatins.


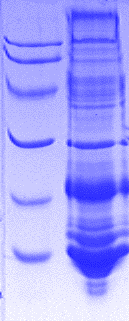


14.4

20.1

30

45

67

94

kDa

*Engystomops*

*pustulosus*


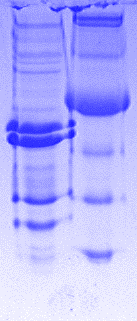


*Leptodactylus*

*fuscus*

*Limnodynastes*

*peronii*

**Supplementary Figure S6.** Diversity in the protein profiles of frog foam nests. SDS-PAGE with Coomassie blue staining of the protein components of the three species of foam nesting frogs named. Nests of *Polypedates leucomystax* and *Rhacophorus arboea* have also been analysed similarly and were also found to be distinct. Molecular size reference proteins are in the left hand lane.

**References for Electronic Supplementary Material**

Bianchet, M. A., Odom, E. W., Vasta, G. R. & Amzel, L. M. 2002 A novel fucose recognition fold involved in innate immunity. *Nature Structural Biology* 9, 628-634.

Dayhoff, M. O., Schwartz, R. M. & Orcutt, B. C. 1978 A Model of Evolutionary Change in proteins. In *Atlas of Protein and Sequence Structure*, vol. 5, suppl. 3 (ed. M. O. Dayhoff), pp. 345-362: National Biomedical Research Foundation, Washington D.C.
